# Application of Mixed Effects Limits of Agreement in the Presence of Multiple Sources of Variability: Exemplar from the Comparison of Several Devices to Measure Respiratory Rate in COPD Patients

**DOI:** 10.1371/journal.pone.0168321

**Published:** 2016-12-14

**Authors:** Richard A. Parker, Christopher J. Weir, Noah Rubio, Roberto Rabinovich, Hilary Pinnock, Janet Hanley, Lucy McCloughan, Ellen M. Drost, Leandro C. Mantoani, William MacNee, Brian McKinstry

**Affiliations:** 1 Edinburgh Clinical Trials Unit, Usher Institute of Population Health Sciences and Informatics, The University of Edinburgh, Edinburgh, United Kingdom; 2 Edinburgh Lung and the Environment Group Initiative (ELEGI), Centre for Inflammation Research, Queen’s Medical Research Institute, The University of Edinburgh, Edinburgh, United Kingdom; 3 Allergy and Respiratory Research Group, Usher Institute of Population Health Sciences and Informatics, The University of Edinburgh, Edinburgh, United Kingdom; 4 Edinburgh Napier University School of Nursing, Midwifery and Social Care, Edinburgh, United Kingdom; 5 e-Health Research Group, Usher Institute of Population Health Sciences and Informatics, The University of Edinburgh, Edinburgh, United Kingdom; University of Texas at Tyler, UNITED STATES

## Abstract

**Introduction:**

The Bland-Altman limits of agreement method is widely used to assess how well the measurements produced by two raters, devices or systems agree with each other. However, mixed effects versions of the method which take into account multiple sources of variability are less well described in the literature. We address the practical challenges of applying mixed effects limits of agreement to the comparison of several devices to measure respiratory rate in patients with chronic obstructive pulmonary disease (COPD).

**Methods:**

Respiratory rate was measured in 21 people with a range of severity of COPD. Participants were asked to perform eleven different activities representative of daily life during a laboratory-based standardised protocol of 57 minutes. A mixed effects limits of agreement method was used to assess the agreement of five commercially available monitors (Camera, Photoplethysmography (PPG), Impedance, Accelerometer, and Chest-band) with the current gold standard device for measuring respiratory rate.

**Results:**

Results produced using mixed effects limits of agreement were compared to results from a fixed effects method based on analysis of variance (ANOVA) and were found to be similar. The Accelerometer and Chest-band devices produced the narrowest limits of agreement (-8.63 to 4.27 and -9.99 to 6.80 respectively) with mean bias -2.18 and -1.60 breaths per minute. These devices also had the lowest within-participant and overall standard deviations (3.23 and 3.29 for Accelerometer and 4.17 and 4.28 for Chest-band respectively).

**Conclusions:**

The mixed effects limits of agreement analysis enabled us to answer the question of which devices showed the strongest agreement with the gold standard device with respect to measuring respiratory rates. In particular, the estimated within-participant and overall standard deviations of the differences, which are easily obtainable from the mixed effects model results, gave a clear indication that the Accelerometer and Chest-band devices performed best.

## Introduction

The Bland-Altman method of limits of agreement is a well-established method of analysing continuous data to assess how well the measurements produced by two raters or devices agree with each other to the extent that they could be used interchangeably without causing any practical problems.[1–4] Although there is plenty of literature available regarding the standard Bland-Altman limits of agreement method; the literature in the area of mixed effects limits of agreement is less well-developed, particularly in the context of multiple sources of variation. Bland and Altman provided the methodological foundation to applying limits of agreement in the context of repeated measures in their 1999 and 2007 papers on using limits of agreement.[2,3] Both of these papers are heavily cited but neither explicitly consider mixed effects regression modelling. More recently, Olofsen and colleagues have published an informative and detailed summary of advanced Bland-Altman methods in the context of repeated measures, but only include a small section on mixed effects regression methods.[5] For those articles that do describe the methodology, there appears to be inconsistency in the terminology and approaches used. For example, Myles and Cui [6] present a mixed effects methodology that involves using time as a random effect and adjusting for “baseline, mean value for the individual over time, and mean measurement between two methods”.[6] Biancofiore and colleagues [7] cite Myles and Cui [6] and appear to use the same methodology; except they write that “the random effect was chosen to reflect the different intercept and slope for each individual with respect to their change of measures over time”[7] implying that they used a random effect for slope as well as a random intercept, which is apparently different from the method that Myles and Cui used in their example. In contrast, Carstensen and colleagues [8] present a methodology of mixed effects agreement which involves separately estimating the variance components within each method/device. In addition, Zou [9] provides a mathematical presentation of limits of agreement when there are multiple measurements per individual, presenting the limits of agreement as functions of a “harmonic mean of replicates” with the mean bias estimated as the mean of the within-subject means. Notwithstanding the individual concerns we might have with the precise details of each of the methods, which we address in the Discussion, it is not clear how the different methods all relate to each other and which (if any) should be preferred. This paper addresses the gap by describing a clear practical application of mixed effects limits of agreement in a study of devices to measure respiratory rate in people with chronic obstructive pulmonary disease (COPD).

Approximately 328 million people are estimated to be living with COPD worldwide,[10] including at least 3 million in the United Kingdom,[11] many of whom experience exacerbations of the condition which can lead to hospital admissions. Respiratory rate monitoring devices could be used to detect early changes in respiratory rate to identify these exacerbations early, enabling timely treatment which would help slow down deterioration and prevent hospital admissions. As a first step towards identifying which devices (if any) had the potential to detect exacerbations early, we assessed how well the measurements from each of five novel devices agreed with the current gold standard respiratory rate monitor (Oxycon mobile, Carefusion) in people with COPD. This was the first phase of a study in three phases. The study design and results of these other phases are presented in a separate paper.[12] In this paper we focus on the statistical methodology of phase 1 only (concerned with limits of agreement) rather than the clinical findings and interpretation of all three phases as presented in Rubio *et al*. [12].

## Methods

### COPD respiratory rate study example

The COPD Respiratory Rate study was approved by the South East Scotland Research Ethics Committee (references: 13/SS/0114, 13/SS/0206 and 14/SS/0043). Participants gave written informed consent to take part in the study. Respiratory rate was measured in 21 people with a range of severity of COPD living in Scotland using five different monitors: described as Camera, Photoplethysmography (PPG), Impedance, Accelerometer, and Chest-band, according to their mode of action. The measurements were made simultaneously with participants wearing all five monitors at the same time in addition to the current gold standard device. It was thus entirely reasonable and valid for us to make direct comparisons between devices because they were all measured at exactly the same time against the same gold standard device. Participants were asked to perform eleven different activities chosen to be representative of everyday tasks during a laboratory-based standardised protocol of 57 minutes of activities. These were sitting, lying, standing, slow walking, fast walking, sweeping, lifting objects, standing and walking, climbing stairs, treadmill (flat walking), and treadmill (4% slope). The activities were designed to test the devices across the full range of plausible measurements. Not everyone performed exactly the same number of activities because some tasks (e.g. the treadmill task) were too difficult for some participants. Also, the number of valid observations per device varied because some devices experienced more technical problems than others, and some were better than others at capturing respiratory rates during the standardised protocol. Therefore, this is an example of a completely unbalanced study design. Furthermore, activity was a potential source of variability in addition to participants and devices. This study design necessitated the use of an advanced form of limits of agreement analysis that takes into account multiple sources of variability and a completely unbalanced study design.

### Limits of agreement

The procedure to calculate limits of agreement involves first calculating the mean and standard deviation of the paired differences (e.g. differences in respiratory rate measured at the same time in the same participant using two different devices). The standard deviation is then multiplied by the 97.5% quantile of a normal distribution (usually rounded to 2) and we then separately add or subtract this quantity from the calculated mean to give the upper or lower limits respectively. If *m* is the mean of the paired differences and *SD* is the standard deviation, the limits of agreement are calculated as: *m* ± 2 * *SD*. The limits of agreement are expected to include about 95% of future observed differences; in reality they are only *estimates* measured with uncertainty, and so 95% confidence intervals are often computed around the limits of agreement themselves. The limits of agreement must then be interpreted clinically to assess whether the agreement is acceptable or not. Ideally, the acceptable range of agreement should be defined *a priori* to avoid any bias in this decision.[4]

### The independence assumption

Limits of agreement methodology can be applied regardless of whether one of the devices or raters is a gold-standard. However, a necessary assumption of the method is that the observations are independent. When this assumption is violated, for example when we have multiple values recorded per individual, then it is necessary to use a repeated measures version of the method [2,3] to generate appropriate limits otherwise the limits will be too narrow.

### Justification for treating participants as random

The 2007 article by Bland & Altman offers a step-by-step guide for applying the methodology in the case of multiple values per individual.[3] They present separate methods for whether the “true value varies” or whether the “true value is constant”.[3] In this article we only consider the “true value varies” method because it is most relevant in the context of measuring respiratory rate, which clearly varies, as do many other physiological variables such as blood pressure, cardiac output, and HbA1c to name just a few. In brief, this method involves obtaining estimates for the between-participant and within-participant variances based on an analysis of variance (ANOVA) table and then taking the square root of the sum of the variances in order to obtain an appropriate standard deviation to be used in the standard limits of agreement formula.[3] Participants are regarded as fixed effects in this methodology which means that we treat them as consisting of the entire population of interest and do not describe them as coming from a distribution of a wider population of COPD patients. In contrast, it is more natural in our example to regard participants as random effects, and assume they are a random sample from a wider population of COPD patients. This maximises the generalisability of the results to the true population of interest (e.g. all COPD patients). Furthermore, a modification of the method is required in cases such as ours, where differences between activities add a further source of variability. Therefore, in our COPD respiratory rate example, we used a mixed effects approach to maximise the generalisability of results to the COPD population while also taking into account the data structure.

### Statistical methods used

To calculate the mixed effects limits of agreement, we analysed the paired differences of each device compared with the gold-standard using a mixed effects regression model, including participant as a random effect and activity as a fixed effect, using the nlme package [13] in R software version 3.2.3 [14]. If *Y*_*ij*_ represents the *j*th paired difference in respiratory rate between devices for patient *i* doing the *k*th activity, then the paired differences are modelled in the form:

$$Y_{ijk}=\alpha +r_{i}+\beta_{k}+e_{ij},$$

$$r_{i}\sim N\left(0,\sigma_{r}^{2}\right),\;\;\;e_{ij}\sim N\left(0,\sigma_{e}^{2}\right),$$

where *α* is the constant intercept term, *r*_*i*_ is the random effect of the *i*th patient, *β*_*k*_ is the fixed effect of the *k*th activity, and *e*_*ij*_ is the error for paired difference *j* on the *i*th patient.

However, to generate an appropriately weighted estimate of the mean bias we fitted a separate regression model only including a constant term and random effect for participant (i.e. without adjusting for activity). Using the same notation as above, this random effects model was of the form:

$$Y_{ij}=\alpha +r_{i}+e_{ij},$$

$$r_{i}\sim N\left(0,\sigma_{r}^{2}\right),\;\;\;e_{ij}\sim N\left(0,\sigma_{e}^{2}\right),$$

where *α* is the mean bias of interest. This model enabled us to correctly weight across the various activities while still retaining a population-level interpretation as per random effects analysis. Thus we used separate regression models to calculate the mean bias and limits of agreement. Further consideration of this topic is provided in the Discussion section. Example R code for computing the basic limits of agreement is included in the S1 File. The estimated between-participant variance and the within-participant/activity variance were both extracted and then summed to create a total variance for all observations. We only needed to use a simple sum rather than a more complicated formula to combine the variances because the model assumes independence between the random effects and residual error terms. The square root of this total variance gives an estimate of the standard deviation for use in the conventional Bland-Altman limits of agreement formula. Activity was included in the model as a fixed effect so that the limits of agreement would be *adjusted for* activity. However, variability across the different activities was not included in the total variance formula because the amount of data recorded per activity varied across devices: some devices were better than others at capturing movement data thereby potentially biasing any variance estimate for activity.

In calculating the mixed effects limits of agreement we assumed that participants were a representative random sample from the overall population of COPD patients, and that the random participant effects *r*_*i*_ were normally distributed. We also assumed constant mean bias *α* and model residuals *e*_*ij*_ that were independent and normally distributed with a constant variance. It is important to check these assumptions since any violation of the assumptions may lead to biased variance components. Bland-Altman plots provide a quick visual check of these assumptions.[2] For the camera device the appropriateness of the constant mean bias assumption was in doubt because the mean bias appeared to be greater for smaller respiratory rate measurements than for higher ones. In this case, we could have adjusted for the average respiratory rate in our models to produce limits of agreement that better reflect the data as suggested by Bland & Altman[2], but this would have meant that we would lose the consistency of modelling across devices and hence comparison between devices would have been compromised. Besides, as Bland & Altman[2] note, the limits of agreement will be appropriately widened by the violation of this assumption, so “would not lead to the acceptance of poor methods of measurement”.[2]

Further checks of the model assumptions were conducting using (i) plots of the standardized residuals against fitted values, (ii) Q-Q plots of the residuals, and (iii) Q-Q plots of the random effect predictions. There was some evidence of violation of assumptions for all devices except for Impedance. However, after removing clear outliers in the model residuals, model assumptions were found to be valid for all device comparisons. S4 File provides an illustration of how outliers were determined, using the Accelerometer device analysis as an example. For each mixed effects model, Q-Q plots of the residuals were visually examined to identify any clear outliers that interfered with the model fit. Even though removing outliers improved the model fit in general, this practice is anti-conservative and we lose the consistency in modelling across the devices, and so we report both the original model and the model after removing outliers in this paper. Besides, with many observations per participant, the mixed effects model is expected to be reasonably robust to outliers in this case. If, as is likely, the outliers occurred due to incorrectly fitting devices, then it seems reasonable to report both the model results assuming well-fitting devices, and also report model results that are more pragmatic, reflecting their use in daily life.

Ninety-five per cent confidence intervals were constructed around the standard deviations using a parametric bootstrap-t (studentized pivotal) procedure [5, 15] and using an approximate estimate of the variance of the standard deviation of the differences calculated based on the formulae presented in Olofsen et al. [5]. (We noticed that there is an error in the formula shown in Olofsen et al. [5] for calculating the variance of a standard deviation from the variance of a variance: the denominator should be the expected value of the variance.) In order to take into account the two levels of variability, we used “the parametric random effects bootstrap coupled with residual bootstrap” method of resampling as described in Thai et al. [16] to perform bootstrap resampling on both the random effects and the residuals.[16] The same parametric bootstrap-t method was used to construct confidence intervals around the revised mixed effects limits of agreement. A total of 1999 bootstrap resamples were used in each bootstrap procedure as recommended by Carpenter & Bithell.[15] The maximum positive and negative differences were also reported for each of the devices.

For comparative purposes, the resulting limits-of-agreement were compared to those obtained from a fixed effects approach based on an extension of the ANOVA method presented in Bland & Altman[3] for when the “true value varies”. The extension of the Bland-Altman ANOVA method involved treating the activities as another “between-subjects” factor in the ANOVA procedure, but then ignoring the specific results of this factor when calculating the limits of agreement.

## Results

**Fig 1:**
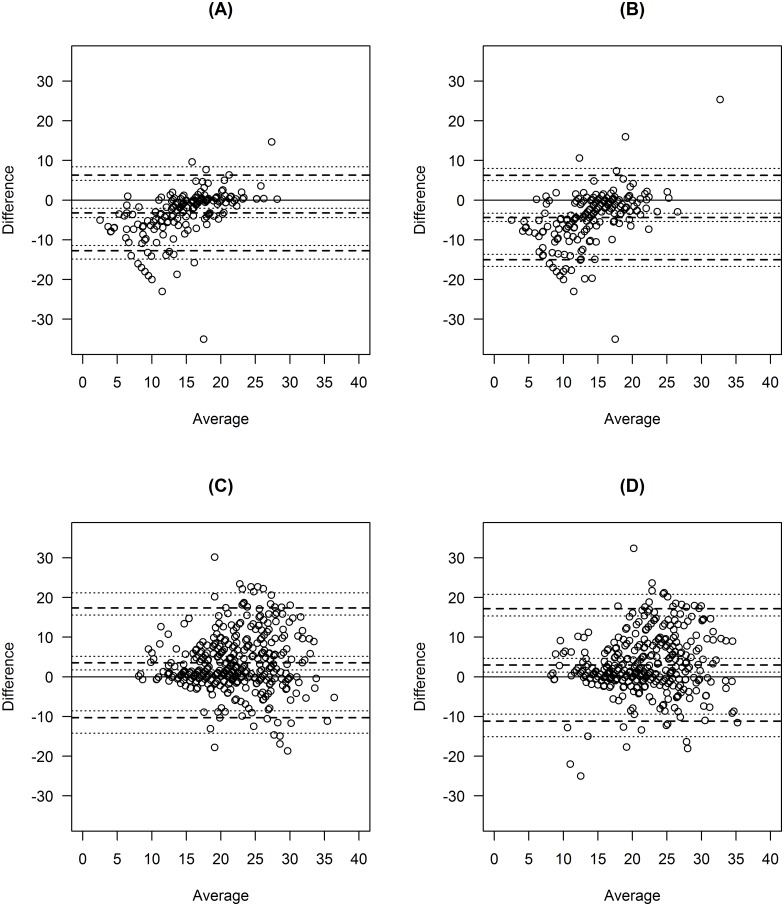
Bland-Altman Plots showing the paired differences against the average for two devices (Camera and PPG). Mean bias and limits of agreement are shown by the dashed lines, while confidence intervals are shown by the dotted lines. (A) Camera: rate per second. (B) Camera: rate per minute. (C) PPG: raw. (D) PPG: median filtered.

**Fig 2:**
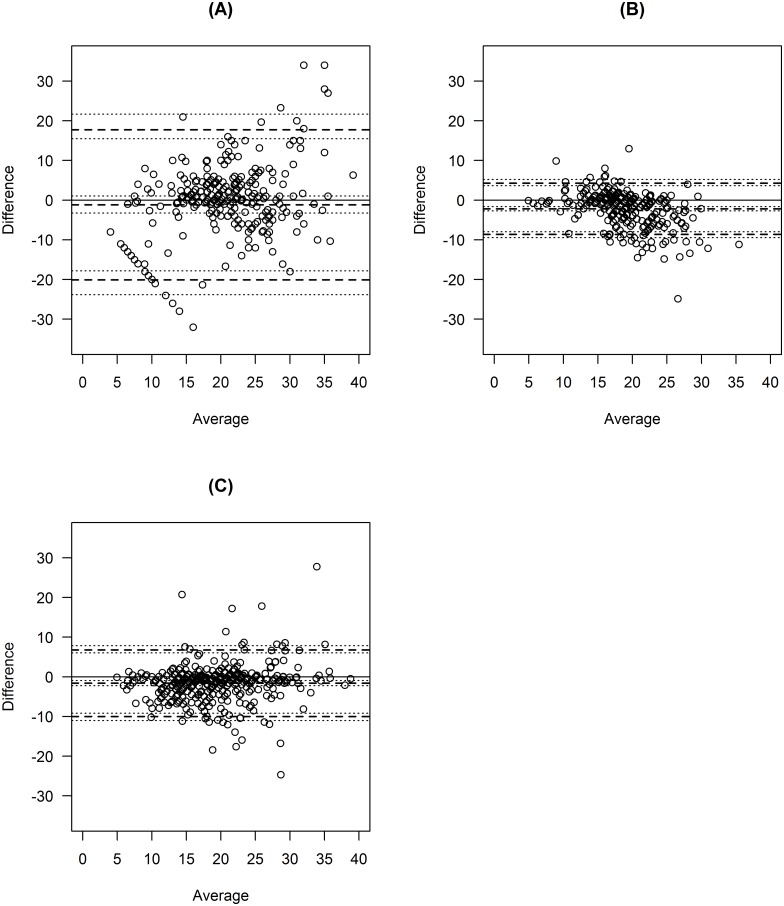
Bland-Altman Plots showing the paired differences against the average for three devices (Impedance, Accelerometer, Chest-band). Mean bias and limits of agreement are shown by the dashed lines, while confidence intervals are shown by the dotted lines. (A) Impedance. (B) Accelerometer. (C) Chest-band.

A total of 21 participants were recruited, but one participant recorded no observations on two devices (see Table 1). An average of 302 valid respiratory rate measurements were recorded across all participants per device (range 192 to 385). Bland-Altman plots were produced showing the paired differences in respiratory rate measurements against the average (see Figs 1 and 2), with mixed-effects limits of agreement and corresponding 95% confidence intervals superimposed on the plots.

**Table 1:** 95% limits of agreement results for the respiratory rate measurements for each of the devices compared to the gold standard measure.

Device	No. of participants(total valid differences/ total after removing outliers)	Mean bias (Fixed effects 95% LoA)	Mean bias (Mixed effects 95% LoA)	Mean bias (REVISED mixed effects 95% LoA*)
Camera (rate per second)	21 (192/188)	-3.32 (-13.35 to 6.72)	-3.21 (-12.71 to 6.30)	-3.15 (-11.54 to 5.24)
Camera (rate per minute)	21 (192/188)	-4.43 (-15.54 to 6.69)	-4.35 (-14.98 to 6.28)	-4.43 (-13.13 to 4.26)
PPG (raw)	21 (378/377)	3.53 (-10.30 to 17.35)	3.53 (-10.30 to 17.35)	3.46 (-10.06 to 16.98)
PPG (median filtered)	21 (378/376)	3.01 (-11.16 to 17.17)	3.01 (-11.17 to 17.19)	3.02 (-10.53 to 16.57)
Impedance	20 (304/304)	-1.17 (-20.07 to 17.73)	-1.18 (-20.07 to 17.72)	-1.18 (-20.07 to 17.72)
Accelerometer	20 (284/282)	-2.18 (-8.74 to 4.38)	-2.18 (-8.63 to 4.27)	-2.14 (-7.91 to 3.63)
Chest-band	21 (385/384)	-1.61 (-9.99 to 6.78)	-1.60 (-9.99 to 6.80)	-1.67 (-9.64 to 6.30)

Mean bias = Average difference; LoA = Limits of Agreement.

*Outliers removed and emphasis is placed on model fitting rather than consistency in methods across devices.

Table 1 shows the numerical values of limits of agreement calculated based on (i) a fixed effects ANOVA method, (ii) a mixed effects model using all possible data, and (iii) a mixed effects model after removing outliers. Only minor differences were seen between the three sets of models. The mixed effects method produced slightly narrower limits of agreement in our case, but this will not always be true in general. Figs 1 and 2 show the mixed effects mean bias and the mixed effects limits of agreement when including outliers.

Table 2 shows within-participant and total (combined) standard deviations of the differences for both the original mixed effects model including outliers, and a revised mixed effects analysis with outliers removed. In this table, the total standard deviation represents the square root of the sum of the within-participant and between-participant variances. Table 3 shows the corresponding 95% bootstrap confidence intervals around the limits of agreement for both sets of mixed effects models.

**Table 2:** Comparison of the variabilities of differences across devices.

Device	Original Mixed Effects Model				Revised Mixed Effects Model*			
	Within- participant SD	Combined SD	Maximum negative difference	Maximum positive difference	Within- participant SD	Combined SD	Maximum negative difference	Maximum positive difference
Camera (rate per second)	4.25(3.87 to 4.79)	4.85(4.44 to 5.76)	35.00	14.71	3.92 (3.58 to 4.44)	4.28 (3.93 to 4.94)	35.00	9.65
Camera (rate per minute)	5.18 (4.74 to 5.86)	5.42 (4.97 to 6.15)	35.00	10.63	4.31 (3.95 to 4.89)	4.44 (4.07 to 4.97)	35.00	10.63
PPG (raw)	6.02 (5.64 to 6.53)	7.05 (6.61 to 8.60)	18.66	30.19	5.85(5.46 to 6.37)	6.90 (6.45 to 8.26)	18.66	23.44
PPG (median filtered)	6.20 (5.81 to 6.77)	7.23 (6.76 to 8.63)	25.00	32.39	5.84 (5.46 to 6.36)	6.91 (6.44 to 8.29)	22.00	23.65
Impedance	8.94 (8.29 to 9.84)	9.64 (8.97 to 10.91)	32.00	34.00	8.94(8.29 to 9.84)	9.64(8.97 to 10.91)	32.00	34.00
Accelerometer	3.23 (3.00 to 3.59)	3.29 (3.05 to 3.62)	24.84	12.99	2.85(2.64 to 3.15)	2.94(2.73 to 3.25)	14.82	12.99
Chest-band	4.17(3.91 to 4.53)	4.28 (4.02 to 4.65)	24.65	27.80	3.98(3.73 to 4.33)	4.07(3.81 to 4.42)	24.65	20.76

*Outliers removed and emphasis is given to model fitting rather than consistency in methods across devices.

**Table 3:** 95% confidence intervals around each of the limits of agreement for the original mixed effects method (results after removing outliers are shown in italics for comparison).

Device	Mean bias	95% repeated differences LoA	95% bootstrap CI of lower limit	95% bootstrap CI of upper limit
Camera (rate per second)	-3.21	-12.71 to 6.30	-14.84 to -11.42	5.00 to 8.39
	*-3*.*15*	*-11*.*54 to 5*.*24*	*-13*.*20 to -10*.*40*	*4*.*10 to 6*.*80*
Camera (rate per minute)	-4.35	-14.98 to 6.28	-16.70 to -13.65	4.96 to 8.00
	*-4*.*43*	*-13*.*13 to 4*.*26*	*-14*.*54 to -11*.*95*	*3*.*16 to 5*.*65*
PPG (raw)	3.53	-10.30 to 17.35	-14.23 to -8.58	15.58 to 21.16
	*3*.*46*	*-10*.*06 to 16*.*98*	*-13*.*63 to -8*.*42*	*15*.*11 to 20*.*49*
PPG (median filtered)	3.01	-11.17 to 17.19	-15.10 to -9.39	15.35 to 20.79
	*3*.*02*	*-10*.*53 to 16*.*57*	*-14*.*34 to -8*.*74*	*14*.*80 to 20*.*33*
Impedance*	-1.18	-20.07 to 17.72	-23.84 to -17.81	15.49 to 21.65
Accelerometer	-2.18	-8.63 to 4.27	-9.45 to -7.96	3.62 to 5.21
	*-2*.*14*	*-7*.*91 to 3*.*63*	*-8*.*75 to -7*.*20*	*3*.*01 to 4*.*64*
Chest-band`	-1.60	-9.99 to 6.80	-11.04 to -9.19	6.05 to 7.86
	*-1*.*67*	*-9*.*64 to 6*.*30*	*-10*.*61 to -8*.*88*	*5*.*62 to 7*.*27*

95% confidence intervals were calculated using a parametric bootstrap-t method based on 1999 resamples.

*No outliers

Two devices (Accelerometer and Chest-band) were regarded as having “acceptable” agreement with the gold standard device because their corresponding limits of agreement were within +/- 10 breaths per minute, although ideally for a high level of agreement we were hoping that the limits would fall within +/- 5 breaths per minute. In any case, these two devices showed the narrowest limits of agreement, and this conclusion was robust to the inclusion or exclusion of outliers. This allowed us to select these two devices for further assessment, which involved testing the acceptability and reliability of the devices in a home setting (see Rubio *et al*. [12]).

The Camera and Impedance devices returned 11 and 37 zero observations respectively. It could be argued that since a respiratory rate of zero is impossible, zero values should not be included in the analysis. However, these were real values returned by the devices, and we included them in order to take a conservative “intention-to-treat” approach which is consistent with the use of these devices in real life. As a sensitivity analysis, re-running the mixed effects analysis on all the data with zeros removed, our results were very similar to before and conclusions unchanged (see S5 File).

## Discussion

This article shows how mixed-effects limits of agreement analysis can be applied relatively easily to the comparison of different devices even when there may be multiple or complex sources of variation in the study design. We compared these limits to a fixed effects approach based on Bland & Altman’s true value varies method and the results were similar. Advantages of the mixed effects approach include the potential for stronger inference and greater generalisability of the results to the target population.[17,18] In addition, the mixed effects approach makes it easier to spot outliers in the model diagnostics and assess the sensitivity of the model to those outliers. However, more distributional assumptions are required;[17] and if the number of participants is very small (i.e. fewer than 10) then a fixed effects approach may be preferred due to concerns about the accuracy of the estimated between-participant variance.[18]

For the mixed effects analysis we directly used the paired differences comparing the devices with the gold standard rather than the raw responses recorded on each device. Carstensen and colleagues [8] separately estimated the variance components within each method/device. However, in the case of what they call “linked replicates” (i.e. true value varies) it is not clear why this methodology should be used rather than an analysis based on the differences, since by including the random interaction terms necessary for analysis one has to wonder about how accurately they can be estimated in small datasets.[8] In the literature, methods for “true value varies” and “true value constant” are often presented separately. In the case of respiratory rates, the true value clearly varies; but even if it was nearly constant within participants or activities, it would still make sense to use the same methodology. We would only use a mixed effects methodology based on the raw measurements for each device if we were *certain* that the true value was constant and/or if measurements were recorded on different occasions, precluding the possibility of calculating paired differences. Indeed, Olofsen et al. found that their “modified” true value varies method has identical statistical properties to the true value constant method when the true value is constant.[5]

Myles and Cui [6] present an alternative mixed effects methodology that involves using time as a random effect and adjusting for the mean of each subject over time and the mean difference between two methods at each measurement occasion.[6] Thus they calculate the within-subject standard deviation “after the between-subject variation (agreement between methods) has been taken into account”. [6] Although this methodology may be appropriate for their specific study, we are concerned about the adoption of this approach in general. In the example used by Myles and Cui,[6] they modelled time as a random effect, presumably because there were only seven independent time points and the assumption of normally distributed random effects was reasonable. However, often time is either (i) a completely continuous variable (i.e. with only one observation at each time point), (ii) there is autocorrelation between time points depending on how close they are to each other, or (iii) the time points are non-random and fixed by study design, all of which may make it difficult to satisfy the mixed model assumptions of independent and normally distributed random effects *r*_*i*_ with constant variance $\;\sigma_{r}^{2}$. This is why in general it is easier to justify modelling participants as random effects rather than time. Secondly, when calculating limits of agreement, the consistency of the agreement across different participants (i.e. the between-subject variation) needs to be taken into account, and not simply the within-participant variation in general. Nevertheless, we do agree that there is value in reporting the separate variance components (or the within-participant standard deviation and total standard deviation as we have done) so that we can identify the proportionate contributions of the different sources of variance to the overall limits of agreement. It could be that over time when measuring respiratory rate in COPD patients we are able to get a sense of the mean bias for a given participant and so the within-subject agreement would be of more interest than the total agreement.

Unlike in standard prediction intervals, the standard error of the mean bias is not included in the calculation of the limits of agreement. Instead, confidence intervals around the mean bias separately quantify the uncertainty in this estimate just as they do around each of the limits of agreement. In fact, there is no requirement for the mean to be derived from exactly the same model as is used to compute the limits of agreement. Olofsen and colleagues suggest that either the raw (or grand) mean or the mean of the participant-level means could be computed.[5] Zou opts for calculating the mean of the participant-level means.[9] In our dataset, we have found that calculating the mean of the activity-level means (or the mixed effects model equivalent) results in very strange estimates of the mean bias. For example, for the camera device (rate per minute), the mixed effects mean adjusting for activities is -14.66 compared to -4.35 without adjustment. This is because the number of non-missing respiratory rate readings for each activity varied enormously from 1 to 144 for the camera device and any mean of the activity-level means would incorrectly weight and therefore bias the mean estimate. In particular, observations within activities that have small sample sizes would be given too much weight when calculating the mean bias. The same would be true when calculating the mean of the participant-level means if the number of observations within participants was highly variable and we chose to use a fixed effects analysis. As Olofsen et al. state, the mean bias is more precisely estimated by the raw mean if the between-participant (or between-activity) variance is high.[5] Yet the decision about the type of mean to use is more important than simply a matter of precision—to use the wrong type would cause an incorrectly weighted estimate of the mean bias. This is why we recommend that researchers calculate either the raw mean as Bland & Altman[3] suggest, or the random-effects level mean that appropriately weights across participants and/or activities.

On the other hand, if the number of multiple respiratory rate readings within each participant and activity was the same (i.e. if the problem was completely balanced), then it would not have mattered how we calculated the mean bias. This is because any fixed or random effects estimate of the mean would be equivalent to the raw mean due to equal weighting of observations in this context.[17] However, this was not the case in our study and so we calculated the random effects level mean by fitting a random effects regression model *unadjusted* for activity; while separately calculating the limits of agreement using a mixed effects model *adjusting* for activity. Of course for comparison purposes it matters less which type of mean we use provided that we are consistent in the use of the same type of mean across all the devices.

Hofman and colleagues[19] also identified problems with an incorrectly weighted mean in the context of Bland-Altman plots. If the between-method differences are calculated from unequal numbers of observations within each method group at each time point, then a “mean of the means” estimate will generate an artificial correlation between the differences and the average in a Bland-Altman plot. The solution, say Hofman and colleagues, is to use the raw mean in the Bland-Altman plot which correctly weights the observations. Of course in our example, we only calculate paired differences derived from just two observations, and so this issue was not relevant in our case.

In limits of agreement analysis we must assume a constant level of agreement across the range of measurement. In some of the Bland-Altman plots shown in Figs 1 and 2, the validity of this assumption is in doubt because in some of the plots the variability appears to increase with the average respiratory rate. This could be due to a floor effect whereby it is unlikely that respiratory rate will be below a certain threshold (e.g. 10 breaths per minute) and indeed impossible for it to be below zero. This artificially restricts the range within which the differences between devices must lie for low values of respiratory rate; whereas for high values there is no such restriction. Therefore, the limits of agreement method is best used in situations where the range of measurement is completely unrestricted and where floor and ceiling effects are negligible; but if there is evidence of floor or ceiling effects then this should be borne in mind when interpreting Bland-Altman plots and limits of agreement. When floor or ceiling effects are present, researchers should be aware that acceptable agreement may (at least partly) be a consequence of the restricted range, and may not necessarily reflect the ability of the different methods/devices to agree. Floor and ceiling effects can also lead to non-normality in the outcome differences.[20]

The reason behind outliers in the model residuals or zero values returned by the devices was unknown, but we believe it may have been due to technical issues with some of the devices or problems with device fitting. This was why it was appropriate for us to include results with and without outliers; otherwise if we excluded all outliers and zero values this may give a false impression of the agreement for some devices. Only a few of the outliers in the models residuals can be attributed to zero values produced by the devices; and it could be that most of the other outliers were caused by inaccurate readings by the devices but these may be difficult to detect from simply looking at the raw data.

In our mixed effects model we considered activities as a fixed effect. Instead, we could have considered the activities as random effects, and this would have enabled us to immediately obtain an estimate of the variability between activities, but the fixed effects assumption for the activities made more sense in this context than assuming the activities were a random sample from a larger population.

The MOVER method developed by Zou [9] or the exact two-sided tolerance limits proposed by Carkeet and Goh [20] could have been used to compute the 95% confidence intervals for the limits of agreement, but both methods depend heavily on the normality assumption and so we opted for performing a parametric bootstrap-t method instead as proposed by Olofsen et al. [9] In situations where we can be confident about the normality of the data, then the MOVER or tolerance limits methods may give improved confidence intervals compared to the parametric bootstrap-t method.[9] It is not appropriate to use the same approximate confidence intervals as for the standard limits of agreement method due to poor coverage.[20, 21].

To encourage future applications of the mixed effects limits of agreement method, the full R code we used is provided in the S2 File. Note that this code was tailored to our particular study design: in other studies there may not be any additional sources of variability and therefore adjustment for this is not needed in the modelling procedure; or the study design may be more complex with multiple levels of variability (e.g. for studies assessing agreement within wards within hospitals within geographical regions). When faced with complex variability structures then it is only necessary to add together the variance components that contribute to the assessment of agreement.

In this article, we showed how the mixed effects limits of agreement method was ideally suited to answer the question of which device had the strongest agreement with the gold standard with respect to measuring respiratory rates in COPD patients. The superiority of the limits of agreement method over alternatives such as calculating correlation coefficients has been discussed elsewhere.[2,4, 22, 23] For completeness, we presented the full limits of agreement with confidence intervals, but the real basis for decision-making was the within-participant and total overall standard deviations of the differences, which allowed us to easily rank the devices to find out which performed best. This methodology is ideal for use in the situation where we are interested in comparing the agreement of raters or devices recording continuous measurements. Repeated measurements should be encouraged in agreement studies because they allow us to quantify the agreement of measurements within the same subject and then compare this with the overall agreement.[2] The methodology involved is relatively straightforward to apply and should present little or no barrier to analysing repeated measures data in practice.

## Supporting Information

S1 File Basic R software code to compute 95% mixed effects limits of agreement. (DOCX)

S2 File Comprehensive R software code to compute Bland-Altman plots, 95% mixed effects limits of agreement, and 95% parametric bootstrap-t confidence intervals. (DOCX)

S3 File The COPD Respiratory dataset used in the analysis (de-identified for public sharing). (XLSX)

S4 File Example of how outliers were determined using QQ-plots (the Accelerometer device). (DOCX)

S5 File Mixed effects limits of agreement results after excluding zero respiratory rate observations. (DOCX)
