# Supplementary material for: Application of Mixed Effects Limits of Agreement in the Presence of Multiple Sources of Variability: Exemplar from the Comparison of Several Devices to Measure Respiratory Rate in COPD Patients
Source: PLoS One. 2016 Dec 14;11(12):e0168321. doi: 10.1371/journal.pone.0168321 (PMC5156413; doi:10.1371/journal.pone.0168321)
Supplement: S1 File — (DOCX) [file pone.0168321.s001.docx]

**####################### S1 Appendix ################################**

**#Warning: The following R code is provided as an example only.**

**#Modication of the code and/or testing may be required.**

**#### The following code assumes that the paired differences between #### raters/devices are stored in ‘Diff’, the different activities #### are stored in ‘Activity’, patient identifier is in ‘Patientid’, #### and all these variables are contained in ‘Data’**

**# Install the “nlme” package and then load it into the R console #using ‘library’**

**library(nlme)**

**# Main model**

**res<-lme(Diff~as.factor(Activity),random=~1|Patientid,**

**correlation=corCompSymm(form=~1|Patientid),data=Data,na.action=na.omit)**

**summary(res)**

**withinsd<-as.numeric(VarCorr(res)[2,2])**

**betweensd<-as.numeric(VarCorr(res)[1,2])**

**totalsd<-sqrt(as.numeric(VarCorr(res)[1,1])+as.numeric(VarCorr(res)[2,1]))**

**# Model to extract appropriately weighted mean and standard error**

**res2<-lme(Diff~1,random=~1|Patientid,**

**correlation=corCompSymm(form=~1|Patientid),data=Data,na.action=na.omit)**

**mean<-summary(res2)$tTable[1,1]**

**se<-summary(res2)$tTable[1,2]**

**# 95% Limits of agreement**

**low<-mean-1.96*totalsd**

**upper<-mean+1.96*totalsd**

**cat(“95% Limits of Agreement are from”,low,”to”,upper,”\n”)**
