# Supplementary material for: Application of Mixed Effects Limits of Agreement in the Presence of Multiple Sources of Variability: Exemplar from the Comparison of Several Devices to Measure Respiratory Rate in COPD Patients
Source: PLoS One. 2016 Dec 14;11(12):e0168321. doi: 10.1371/journal.pone.0168321 (PMC5156413; doi:10.1371/journal.pone.0168321)
Supplement: S2 File — (DOCX) [file pone.0168321.s002.docx]

**####################### S2 Appendix ################################**

**#Warning: The following R code is provided as an example only.**

**#Modication of the code and/or testing may be required.**

**####################################################################**

**# Function bootMixedfunction performs the mixed effects modelling**

**# and outputs relevant statistics**

**####################################################################**

**###### INPUTS:**

**# datainput = Bootstrap dataset**

**###### OUTPUTS:**

**# Lower and upper 95% LoA, Between subjects SD, Total SD, Mean bias, # standard error of the mean bias**

**####################################################################**

**bootMixedfunction<-function(datainput){**

**library(nlme)**

**resb<-lme(bootdiffsim~as.factor(Activity),random=~1|Patientid,**

**correlation=corCompSymm(form=~1|Patientid),data=datainput,na.action=na.omit)**

**summary(resb)**

**bootwithinsd<-as.numeric(VarCorr(resb)[2,2])**

**bootbetweensd<-as.numeric(VarCorr(resb)[1,2])**

**boottotalsd<-sqrt(as.numeric(VarCorr(resb)[1,1])+as.numeric(VarCorr(resb)[2,1]))**

**resb2<-lme(bootdiffsim~1,random=~1|Patientid,**

**correlation=corCompSymm(form=~1|Patientid),data=datainput,na.action=na.omit)**

**mean<-summary(resb2)$tTable[1,1]**

**se<-summary(resb2)$tTable[1,2]**

**low<-mean-1.96*boottotalsd**

**upper<-mean+1.96*boottotalsd**

**out<-cbind(low,upper,bootwithinsd,bootbetweensd,boottotalsd,mean,se)**

**out**

**}**

**##################################################################**

**#Function bootfn performs the parametric bootstrap method for the #95% CI and calls ‘bootMixedfunction’**

**#################################################################**

**###### INPUTS:**

**# data1 = Formatted dataset (see ‘parabootstraptLOA’)**

**# nboot = Number of bootstrap resamples**

**# origres = Mixed effects regression model object**

**###### OUTPUTS:**

**# Bootstrap estimates of the lower and upper 95% LoA, Between #subjects SD, Total SD, Mean bias, standard error of the mean bias**

**####################################################################**

**bootfn<-function(data1,nboot,origres){**

**fits<-predict(origres,level=0)**

**newREpred<-rnorm(length(levels(as.factor(data1$Patientid))),**

**0,as.numeric(VarCorr(origres)[1,2]))**

**nperppt<-by(data1$diff,INDICES=data1$Patientid,FUN=function(x) sum(is.na(x)=="FALSE"))**

**newREdata<-rep(newREpred,times=as.numeric(nperppt))**

**bootlow<-NULL**

**bootupper<-NULL**

**bootwithinsd<-NULL**

**bootbetweensd<-NULL**

**boottotalsd<-NULL**

**bootmean<-NULL**

**bootse<-NULL**

**for(j in 1:nboot){**

**bootdiffsim<-fits+newREdata+rnorm(length(fits),0,**

**as.numeric(VarCorr(origres)[2,2]))**

**bootdata<-data.frame(data1,bootdiffsim)**

**bootMixedfunction(bootdata)**

**bootlow[j]<-bootMixedfunction(bootdata)[1]**

**bootupper[j]<-bootMixedfunction(bootdata)[2]**

**bootwithinsd[j]<-bootMixedfunction(bootdata)[3]**

**bootbetweensd[j]<-bootMixedfunction(bootdata)[4]**

**boottotalsd[j]<-bootMixedfunction(bootdata)[5]**

**bootmean[j]<-bootMixedfunction(bootdata)[6]**

**bootse[j]<-bootMixedfunction(bootdata)[7]**

**}**

**output<-cbind(bootlow,bootupper,bootwithinsd,bootbetweensd,boottotalsd,bootmean,bootse)**

**names(output)<-c(bootlow,bootupper,bootwithinsd,bootbetweensd,boottotalsd,bootmean,bootse)**

**output**

**}**

**##################################################################**

**#Function ‘parabootstraptLOA’ processes the parametric bootstrap #estimates generated by function ‘bootfn’ to construct 95% #bootstrap-t confidence intervals**

**###### INPUTS:**

**# nboot = Specify number of bootstrap resamples**

**# dataorig0 = Original dataset**

**# Contrastdevice = vector of responses from the test monitor**

**# Vector = Optional variable with a vector of all outliers**

**# Seed = Seed for the random sampling**

**###### OUTPUTS:**

**# Bland-Altman plot with lines representing 95% LoA and 95% CI, 95% # bootstrap confidence intervals, within-patient SD with 95% CI, #between-patient SD with 95% CI, and other relevant statistics,**

**####################################################################**

**parabootstraptLOA<-function(nboot,dataorig0,Contrastdevice,vector,seed=10){**

**set.seed(seed)**

**# Gold standard device**

**gold<-dataorig0$RRox**

**# Calculate the paired differences**

**diff<-Contrastdevice-gold**

**# Calculate the average**

**average<-(Contrastdevice+gold)/2**

**dataorig02<-data.frame(dataorig0,gold,diff,average)**

**#####Code for removing outliers if required.**

**##### Warning!: delete “[-vector]” from line below if you do not ##### wish to remove outliers.**

**dataorig03<-dataorig02[-vector,]**

**dataorig<-dataorig03[is.na(dataorig03$diff)==0,]**

**library(nlme)**

**resa<-lme(diff~as.factor(Activity),random=~1|Patientid,**

**correlation=corCompSymm(form=~1|Patientid),data=dataorig,na.action=na.omit)**

**summary(resa)**

**withinsd<-as.numeric(VarCorr(resa)[2,2])**

**betweensd<-as.numeric(VarCorr(resa)[1,2])**

**totalsd<-sqrt(as.numeric(VarCorr(resa)[1,1])+as.numeric(VarCorr(resa)[2,1]))**

**resa2<-lme(diff~1,random=~1|Patientid,**

**correlation=corCompSymm(form=~1|Patientid),data=dataorig,na.action=na.omit)**

**meana<-summary(resa2)$tTable[1,1]**

**sea<-summary(resa2)$tTable[1,2]**

**lowa<-meana-1.96*totalsd**

**uppera<-meana+1.96*totalsd**

**obsres<-c(lowa,uppera,withinsd,betweensd,totalsd,meana,sea)**

**withinvar<-withinsd^2**

**betweenvar<-betweensd^2**

**totalvar<-totalsd^2**

**varm<-sea^2**

**store<-bootfn(dataorig,nboot,resa)**

**slist<-NULL**

**slist<-as.numeric(by((is.na(dataorig$diff)==0),INDICES=dataorig$Patientid,FUN=sum))**

**n<-length(slist[slist!=0])**

**sumM<-sum(slist)**

**slist2<-NULL**

**for(i in 1:n){slist2<-c(slist2,slist[i]^2)}**

**sumMsquared<-sum(slist2)**

**lambda<-((sumM^2)-sumMsquared)/((n-1)*sumM)**

**lambda**

**# bootstrap replicates#**

**# bootlower loa #**

**b1<-store[,1]**

**# bootupper loa #**

**b2<-store[,2]**

**# boot within sd #**

**b3<-store[,3]**

**# boot between sd #**

**b4<-store[,4]**

**# boot total sd #**

**b5<-store[,5]**

**# boot mean #**

**b6<-store[,6]**

**# boot se #**

**b7<-store[,7]**

**# boot within var #**

**b8<-b3^2**

**# boot between var #**

**b9<-b4^2**

**# boot total var #**

**b10<-b5^2**

**var1star<-( 2*( ((1-1/lambda)*b8)^2 )/(sumM-n) )+ ( 2*((b8/lambda+b9)^2)/(n-1) )**

**var2star<- 0.25*(var1star/b10)**

**var3star<-(b7^2)+(1.96^2)*var2star**

**var1<-( 2*( ((1-1/lambda)*withinvar)^2 )/(sumM-n) )+ ( 2*((withinvar/lambda+betweenvar)^2)/(n-1) )**

**var2<- 0.25*(var1/totalvar)**

**var3<-varm+(1.96^2)*var2**

**### Lower bootstrap limits**

**teeb1<-(b1-lowa)/var3star**

**steeb1<-sort(teeb1)**

**lowb1<-lowa-steeb1[0.975*(nboot+1)]*var3**

**highb1<-lowa-steeb1[0.025*(nboot+1)]*var3**

**### Upper bootstrap limits**

**teeb2<-(b2-uppera)/var3star**

**steeb2<-sort(teeb2)**

**lowb2<-uppera-steeb2[0.975*(nboot+1)]*var3**

**highb2<-uppera-steeb2[0.025*(nboot+1)]*var3**

**### Within patient sd**

**var4<-2*(withinvar^2)/(sumM-n)**

**var5<-0.25*(var4/withinvar)**

**var4star<-2*(b8^2)/(sumM-n)**

**var5star<-0.25*(var4star/b8)**

**teeb3<-(b3-withinsd)/var5star**

**steeb3<-sort(teeb3)**

**lowb3<-withinsd-steeb3[0.975*(nboot+1)]*var5**

**highb3<-withinsd-steeb3[0.025*(nboot+1)]*var5**

**### Total sd**

**teeb5<-(b5-totalsd)/var2star**

**steeb5<-sort(teeb5)**

**lowb5<-totalsd-steeb5[0.975*(nboot+1)]*var2**

**highb5<-totalsd-steeb5[0.025*(nboot+1)]*var2**

**### Mean**

**teeb6<-(b6-meana)/b7**

**steeb6<-sort(teeb6)**

**lowb6<-meana-steeb6[0.025*(nboot+1)]*sea**

**highb6<-meana-steeb6[0.975*(nboot+1)]*sea**

**plot(average,diff,ylab="Difference",xlab="Average",ylim=c(-36,36),xlim=c(0,40),xaxp=c(0,40,8),las=1)**

**abline(h=0)**

**abline(h=meana,lty=2,lwd=1.5)**

**abline(h=lowa,lty=2,lwd=1.5)**

**abline(h=uppera,lty=2,lwd=1.5)**

**cat("LoA are", lowa, "to" ,uppera,"\n")**

**cat("Maximum negative difference is",abs(min(dataorig$diff,na.rm=T)),"\n")**

**cat("Maximum positive difference is",max(dataorig$diff,na.rm=T),"\n")**

**cat("Mean bias is",meana,"\n")**

**abline(h=lowb1,lty=3)**

**abline(h=lowb2,lty=3)**

**abline(h=highb1,lty=3)**

**abline(h=highb2,lty=3)**

**abline(h=lowb6,lty=3)**

**abline(h=highb6,lty=3)**

**cat("95% bootstrap confidence interval**

**for the lower limit is",lowb1, "to", highb1,"\n")**

**cat("95% bootstrap confidence interval**

**for the upper limit is",lowb2, "to", highb2,"\n")**

**cat("Within SD is",withinsd,"\n")**

**cat("95% bootstrap confidence interval**

**for the within SD is",lowb3, "to", highb3,"\n")**

**cat("Total SD is",totalsd,"\n")**

**cat("95% BC bootstrap confidence interval**

**for the total SD is",lowb5, "to", highb5,"\n")**

**cat("95% BC bootstrap confidence interval**

**for the mean is",lowb6, "to", highb6,"\n")**

**}**

**####################################################################**

**####################################################################**

**### Read in the data #####**

**dataorig0<-read.table("data.csv",sep=",",fill=T,header=T)**

**names(dataorig0)**

**tiff(filename="Figure1A.tiff",width=19,height=22,units="cm",compression="lzw",res=600)**

**par(mfrow=c(2,2))**

**# Camera: Rate per second ###**

**vector<-c(55,72,112,262)**

**parabootstraptLOA(nboot=1999,dataorig0=dataorig0,Contrastdevice=dataorig0$RRcm1,vector=vector,seed=1859)**

**title("Camera: Rate per second")**

**# Camera: Rate per minute ###**

**vector<-c(55,72,112,262)**

**parabootstraptLOA(nboot=1999,dataorig0=dataorig0,Contrastdevice=dataorig0$RRcm2,vector=vector,seed=1859)**

**title("Camera: Rate per minute")**

**# PPG: Raw ###**

**vector<-19**

**parabootstraptLOA(nboot=1999,dataorig0=dataorig0,Contrastdevice=dataorig0$RRppg1,vector=vector,seed=1860)**

**title("PPG: Raw")**

**# PPG: Median filtered ###**

**vector<-c(8,19)**

**parabootstraptLOA(nboot=1999,dataorig0=dataorig0,Contrastdevice=dataorig0$RRppg2,vector=vector,seed=1861)**

**title("PPG: Median filtered")**

**dev.off()**

**tiff(filename="Figure1B.tiff",width=19,height=22,units="cm",compression="lzw",res=600)**

**par(mfrow=c(2,2))**

**# Impedance ###**

**vector<-2**

**parabootstraptLOA(nboot=1999,dataorig0=dataorig0,Contrastdevice=dataorig0$RRimp,vector=vector,seed=1862)**

**title("Impedance")**

**# Accel ###**

**vector<-c(19,166)**

**parabootstraptLOA(nboot=1999,dataorig0=dataorig0,Contrastdevice=dataorig0$RRacc,vector=vector,seed=1863)**

**title("Accel")**

**# Chest-band ###**

**vector<-261**

**parabootstraptLOA(nboot=1999,dataorig0=dataorig0,Contrastdevice=dataorig0$RRcb,vector=vector,seed=1864)**

**title("Chest-band")**

**dev.off()**
