# Supplementary material for: Application of Mixed Effects Limits of Agreement in the Presence of Multiple Sources of Variability: Exemplar from the Comparison of Several Devices to Measure Respiratory Rate in COPD Patients
Source: PLoS One. 2016 Dec 14;11(12):e0168321. doi: 10.1371/journal.pone.0168321 (PMC5156413; doi:10.1371/journal.pone.0168321)

**Accelerometer device**

**Plot A: Q-Q plot of residual errors (for model including outliers)**


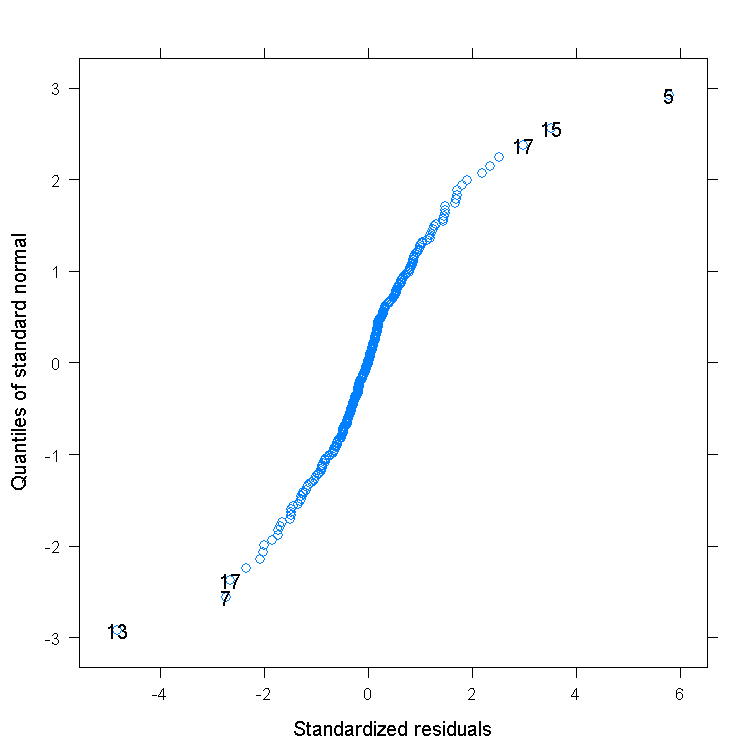


**Plot B: Q-Q plot of residual errors (for model excluding two outliers)**


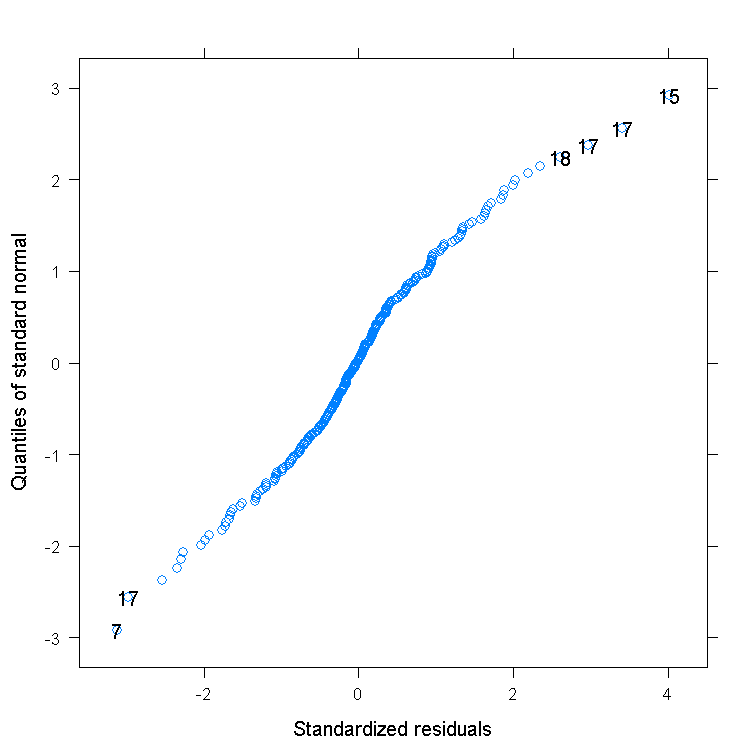


**Plot C: Q-Q plot of residual errors (for model excluding zero observations)**


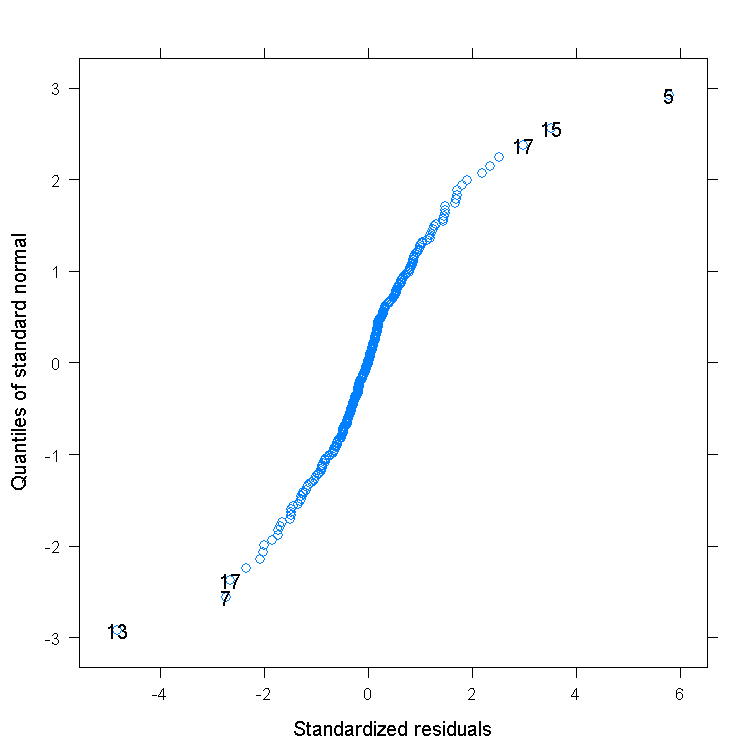

Supplement: S4 File — (DOCX) [file pone.0168321.s004.docx]
