# Supplementary material for: Application of Mixed Effects Limits of Agreement in the Presence of Multiple Sources of Variability: Exemplar from the Comparison of Several Devices to Measure Respiratory Rate in COPD Patients
Source: PLoS One. 2016 Dec 14;11(12):e0168321. doi: 10.1371/journal.pone.0168321 (PMC5156413; doi:10.1371/journal.pone.0168321)
Supplement: S5 File — (DOCX) [file pone.0168321.s005.docx]

**Table S5.1: 95% limits of agreement results for the respiratory rate measurements for each of the devices compared to the gold standard measure, with zero values removed.**

| **Device** | **No. of participants**  **(total valid differences/ total after removing zeros)** | **Mean bias (Mixed effects 95% LoA)** |
| --- | --- | --- |
| Camera (rate per second) | 21 (192/181) | -2.44 (-11.19 to 6.32) |
| Camera (rate per minute) | 21 (192/181) | -3.61 (-13.59 to 6.37) |
| PPG (raw) | 21 (378/378) | 3.53 (-10.30 to 17.35) |
| PPG (median filtered) | 21 (378/376) | 3.16 (-10.58 to 16.89) |
| Impedance | 20 (304/267) | 1.48 (-12.89 to 15.85) |
| Accelerometer | 20 (284/284) | -2.18 (-8.63 to 4.27) |
| Chest-band | 21 (385/385) | -1.60 (-9.99 to 6.80) |

Mean bias = Average difference

LoA = Limits of Agreement

**Table S5.2: Comparison of the variabilities of differences across devices, with zero values removed.**

| **Device** | **Mixed Effects Model after removing zeros** | | | |
| --- | --- | --- | --- | --- |
|  | **Within-** **participant SD** | **Combined SD** | **Maximum negative difference** | **Maximum positive difference** |
| Camera (rate per second) | 3.95 (3.60 to 4.47) | 4.47 (4.10 to 5.27) | 18.71 | 14.71 |
| Camera (rate per minute) | 4.93  (4.51 to 5.59) | 5.09  (4.67 to 5.71) | 19.82 | 25.38 |
| PPG (raw) | 6.02  (5.64 to 6.53) | 7.05  (6.61 to 8.60) | 18.66 | 30.19 |
| PPG (median filtered) | 5.95 (5.56 to 6.50) | 7.01 (6.53 to 8.40) | 18.03 | 32.39 |
| Impedance | 6.63  (6.12 to 7.32) | 7.33  (6.79 to 8.59) | 21.33 | 34.00 |
| Accelerometer | 3.23  (3.00 to 3.59) | 3.29 (3.05 to 3.62) | 24.84 | 12.99 |
| Chest-band | 4.17  (3.91 to 4.53) | 4.28  (4.02 to 4.65) | 24.65 | 27.80 |
